# A Comprehensive Review of the Chemistry, Pharmacokinetics, Pharmacology, Clinical Applications, Adverse Events, and Quality Control of Indigo Naturalis

**DOI:** 10.3389/fphar.2021.664022

**Published:** 2021-05-31

**Authors:** Quan Sun, Jing Leng, Ling Tang, Lijuan Wang, Chaomei Fu

**Affiliations:** ^1^ School of Pharmacy, Chengdu University of Traditional Chinese Medicine, Chengdu, China; ^2^ Department of Pharmacy, Chongqing Hospital of Traditional Chinese Medicine, Chongqing, China; ^3^ Department of Pathology, Chongqing Hospital of Traditional Chinese Medicine, Chongqing, China

**Keywords:** pharmacokinetics, pharmacology, clinical applications, indigo naturalis, adverse events, chemistry

## Abstract

Indigo naturalis (IN), which is derived from indigo plants such as Strobilanthes cusia (Nees) Kuntze, Persicaria tinctoria (Aiton) Spach, and Isatis tinctoria L., has been traditionally used in the treatment of hemoptysis, epistaxis, chest pain, aphtha, and infantile convulsion in China for thousands of years. Clinical trials have shown that the curative effect of IN for psoriasis and ulcerative colitis (UC) is remarkable. A total of sixty-three compounds, including indole alkaloids, terpenoids, organic acids, steroids, and nucleosides, have been isolated from IN, of which indole alkaloids are the most important. Indirubin, isolated from IN, was used as a new agent to treat leukemia in China in the 1970s. Indirubin is also an active ingredient in the treatment of psoriasis. Pharmacological studies have confirmed that IN has inhibitory effects on inflammation, tumors, bacteria, and psoriasis. Indigo, indirubin, tryptanthrin, isorhamnetin, indigodole A, and indigodole C are responsible for these activities. This review provides up-to-date and comprehensive information on IN with regard to its chemistry, pharmacokinetics, pharmacology, clinical applications, adverse events, and quality control. This review may also serve a reference for further research on IN.

## Introduction

Indigo naturalis (IN), also called “*Qingdai*” in Chinese, is a dark blue powder, mass, or particle made from the leaf or stem of Strobilanthes cusia (Nees) Kuntze, Persicaria tinctoria (Aiton) Spach, and Isatis tinctoria L. IN was first recorded in the materia medica book “Yao Xing Lun”, which was compiled during the Tang dynasty of China, approximately 1,400 years ago. It was also recorded in other classics of traditional medicine, such as “Kai Bao Ben Cao,” “Ben Cao Gang Mu,” and “Ben Cao Xin Bian,” which were written in the Song, Ming, and Qing dynasties in China, respectively (Sun X. L. et al., 2020). This herb was described as being cold in property and salty in taste, and it affects the liver and lung. IN was traditionally applied in the treatment of hemoptysis, epistaxis, chest pain, aphtha, and infantile convulsion. In the 1970s, Chinese scholars discovered that IN had an effect on the treatment of cancer. In recent years, IN has shown extraordinary clinical efficacy in the treatment of gastrointestinal and skin diseases. IN has also been widely used in Japan and South Korea (Kyoung et al., 2018; Naganuma et al., 2018). In addition, IN was once proposed by the European Pharmacopoeia Commission for inclusion in Ph.D. Eur (Sluis et al., 2007). Owing to the significant medical value of IN, researchers have carried out many in-depth and comprehensive studies of this substance. To date, sixty-three chemical compounds, including indole alkaloids, terpenoids, organic acids, steroids, and nucleosides, have been isolated from IN and identified. Indirubin, a main active ingredient in IN, was used as a new agent to treat leukemia in China in the 1970s. Pharmacological studies have confirmed that IN has an inhibitory effect on inflammation, tumors, bacteria, and psoriasis. Clinical trials showed that the curative effect of IN on psoriasis and UC was remarkable. However, there are no comprehensive summaries or analyses of these advances. In this review, we provide a comprehensive overview of studies on the chemistry, pharmacokinetics, pharmacology, clinical applications, adverse events, and quality control of IN for further research.

## Materials and Methods

The information related to IN in this review was collected from books, including the Chinese Pharmacopoeia and Traditional Chinese Medicine classics and from literature databases such as PubMed, Science Direct, CNKI (China National Knowledge Infrastructure), Google Scholar, and Baidu Scholar.

### Chemical Compounds

#### Indole Alkaloids

**FIGURE 1:**
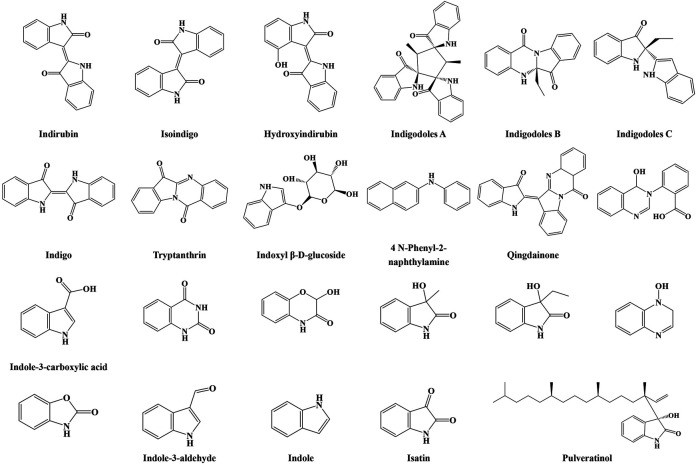
Structures of indole alkaloids from IN.

To date, more than sixty chemical compounds, including indole alkaloids, terpenoids, organic acids, steroids, and nucleosides, have been isolated and identified from IN. Indole alkaloid is one of the main components in IN. A total of twenty-three alkaloids were isolated and investigated. In the 1970s, Wu et al. (1978) first reported the isolation of indirubin and indigo from IN, and these compounds were consistently found by at least four research teams using different methods from 1980 to 2018. Contents of 2% indigo and 0.13% indirubin are the minimum requirements for the quality control of IN in the *Pharmacopeia of the People’s Republic of China*. Li et al. (1983) isolated and identified tryptanthrin, which exhibits antifungal effects against *Microsporum lanosum* and *Trichophyton tonsurans* with a minimal inhibitory concentration of 5 μg/ml. Tryptanthrin also induced apoptosis of K562 cells *in vitro*. In another work (Cheng and Xie, 1984), IN was extracted with ethyl alcohol, and N-phenyl-2-naphthylamine and isoindigo were first identified. In 1985, qingdainone was isolated and identified from IN and showed antimelanoma B1 effects (Zhou and Huang, 1985). IN was extracted with 70% ethanol by reflux and filtered; vacuum drying was applied to dry the filtrate; and the water solution of the crude extract was sequentially fractionated by n-hexane, chloroform, ethyl acetate, and butanol. Then a new indole alkaloid, pulveratinol, was isolated from IN (Kyoung et al., 2018). Crude extract of IN was obtained by four times extraction with methanol; the crude extract was partitioned by ethyl acetate and water; the ethyl acetate–soluble fraction was further fractionated by hexane and 90% methanol; and the aqueous fraction was further fractionated by butanol and water. Indigodoles A, B, and C, which had never been reported, were isolated from IN. Indigodole C significantly inhibited the production of IL-17 in Th17 cells. Moreover, the expression of the IL-17 gene was notably inhibited in a dose-dependent manner by indigodole A (Lee et al., 2019). Other indole alkaloids have also been reported, including isatin, 3-hydroxy-3-methyl oxindole, 3-ethyl-1,3-dihydro-3-hydroxy-2H-indol-2-one, indole-3-carboxylic acid, 2-benzoxazolinone, hydroxyindirubin, indoxyl β-D-glucoside, 2-hydroxy-1,4-benzoxazin-3-one, 3-(2’-carboxyphenyl)-4(3H)-quinazolinol, 4(3H)-quinoxalinol, (1H,3H)-quinazoline-2,4-dione, indole, and indole-3-aldehyde (Li et al., 1983; Wu, 2016; Kyoung et al., 2018; Naganuma et al., 2018). Their structures are shown in Figure 1.

#### Organic Acids

**FIGURE 2:**
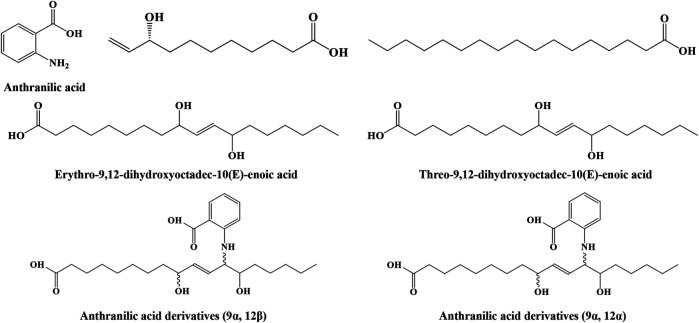
Structures of organic acids from IN.

Two studies (Wu et al., 2016; Kyoung et al., 2018) identified seven organic acids from IN, including anthranilic acid, anthranilic acid derivatives (9α, 12α), anthranilic acid derivatives (9α, 12β), (R)-9-hydroxy-10-undecenoic acid, n-heptadecanoic acid, threo-9,12-dihydroxyoctadec-10(E)-enoic acid, and erythro-9,12-dihydroxyoctadec-10(E)-enoic acid. Anthranilic acid derivatives, (R)-9-hydroxy-10-undecenoic acid, and threo-9,12-dihydroxyoctadec-10(E)-enoic acid, exerted neuroprotective effects on glioma cells. Their structures are shown in Figure 2.

#### Terpenoids

**FIGURE 3:**
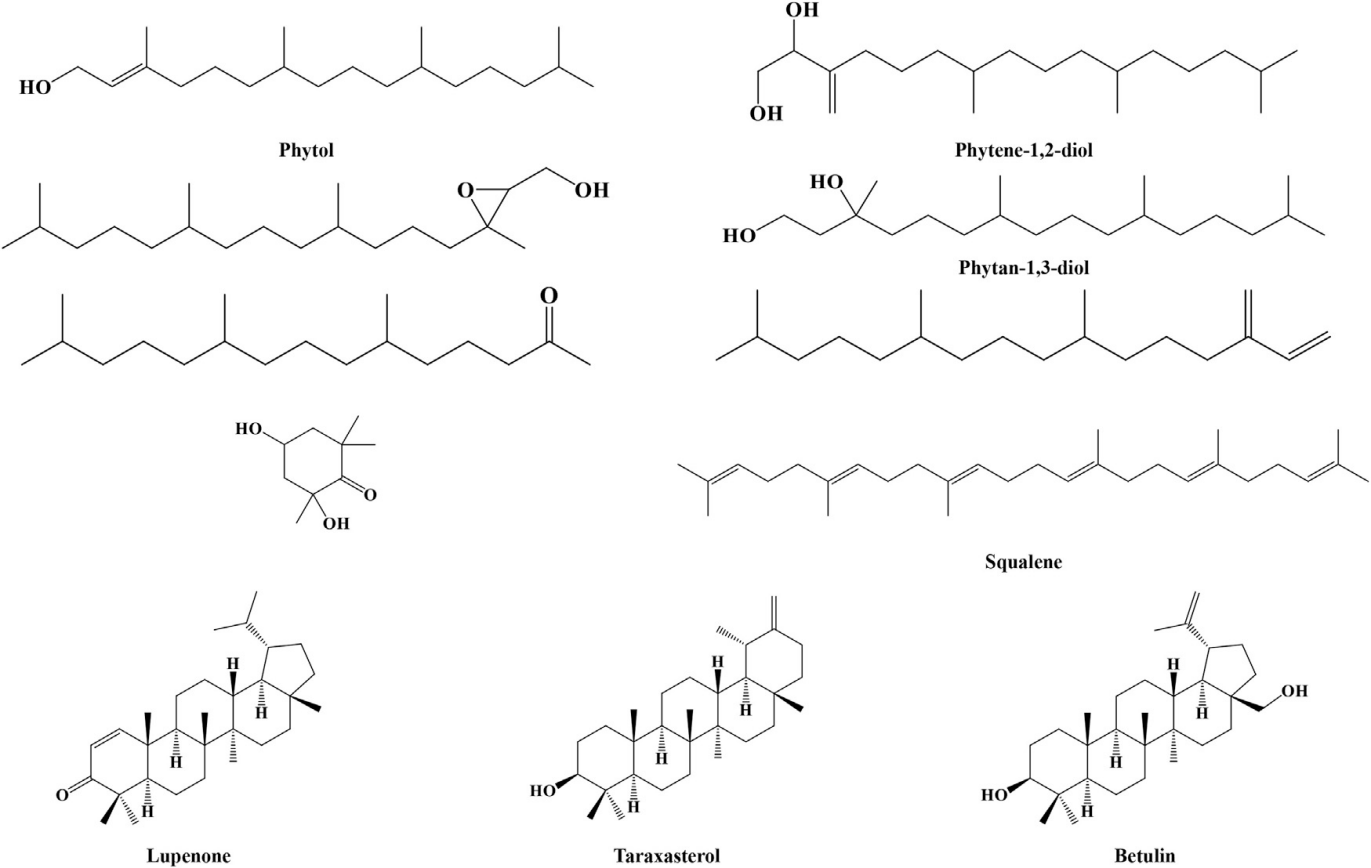
Structures of terpenoids from IN.

Terpenoids in IN include phytol, phytene-1,2-diol, (2R, 3R)-(E)-phytol epoxide, phytan-1,3-diol, 7,11,15-trimethyl-3-methylidene-hexadec-1-ene, 6,10,14-trimethyl-2-pentadecanone, 2,4-dihydroxy-2,6,6-trimethylcyclohexanone, squalene, lupenone, taraxasterol, and betulin, whose structures are shown in Figure 3.

#### Steroids

**FIGURE 4:**
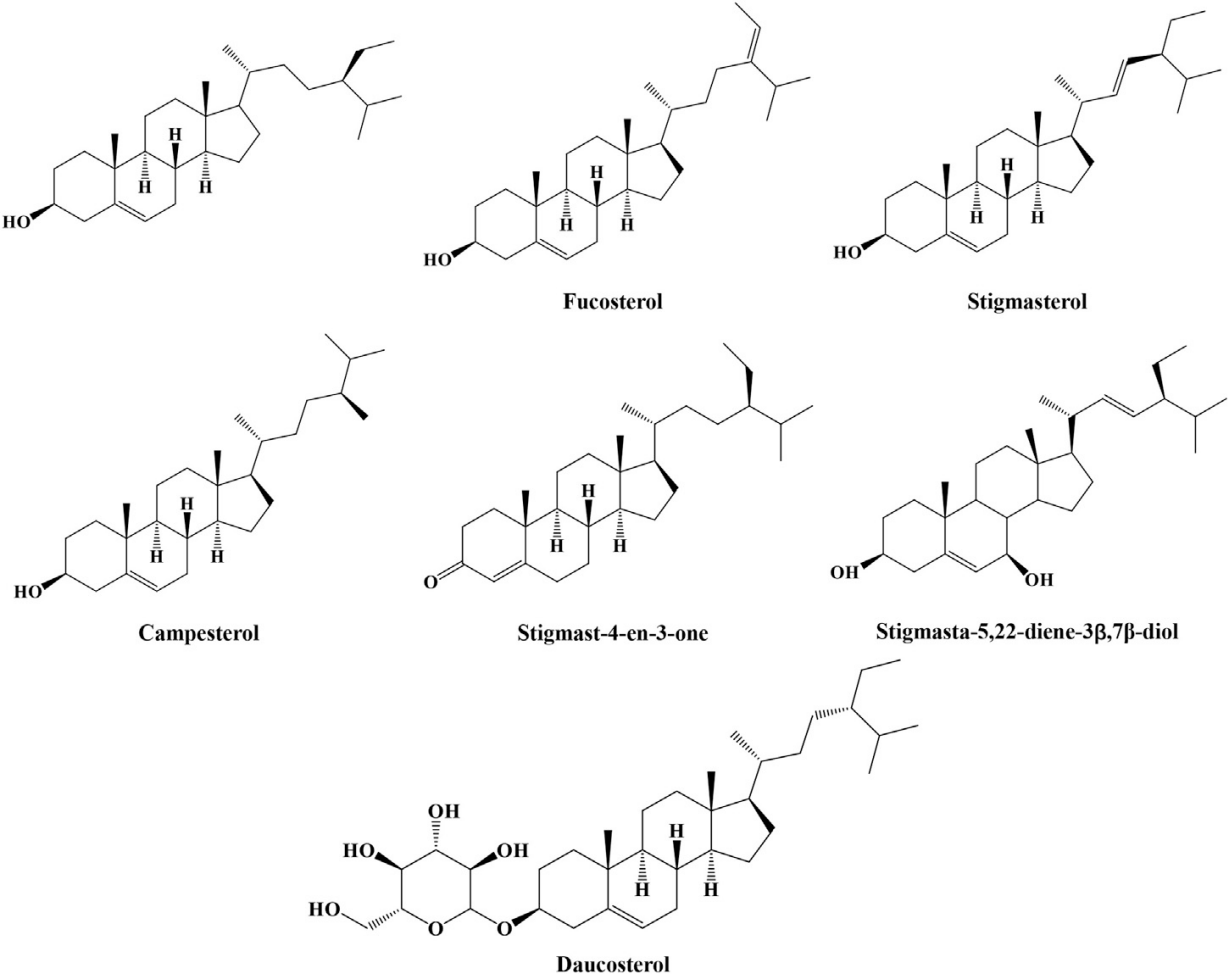
Structures of steroids from IN.

Phytosterols are extensively used in cosmetics, foods, and medicines. Eight steroids have been reported and characterized. In 1984, β-sitosterol was found to exist in IN (Cheng and Xie, 1984). Moreover, IN was extracted by Wu et al. with diethyl ether through ultrasonic extraction for 30 min. Campesterol, stigmasterol, fucosterol, and stigmast-4-en-3-one were isolated and identified. In further studies, stigmasta-5,22-diene-3β, 7β-diol and daucosterol were obtained from IN (Wu et al., 2016; Naganuma et al., 2018). All of these structures are shown in Figure 4.

#### Nucleosides

**FIGURE 5:**
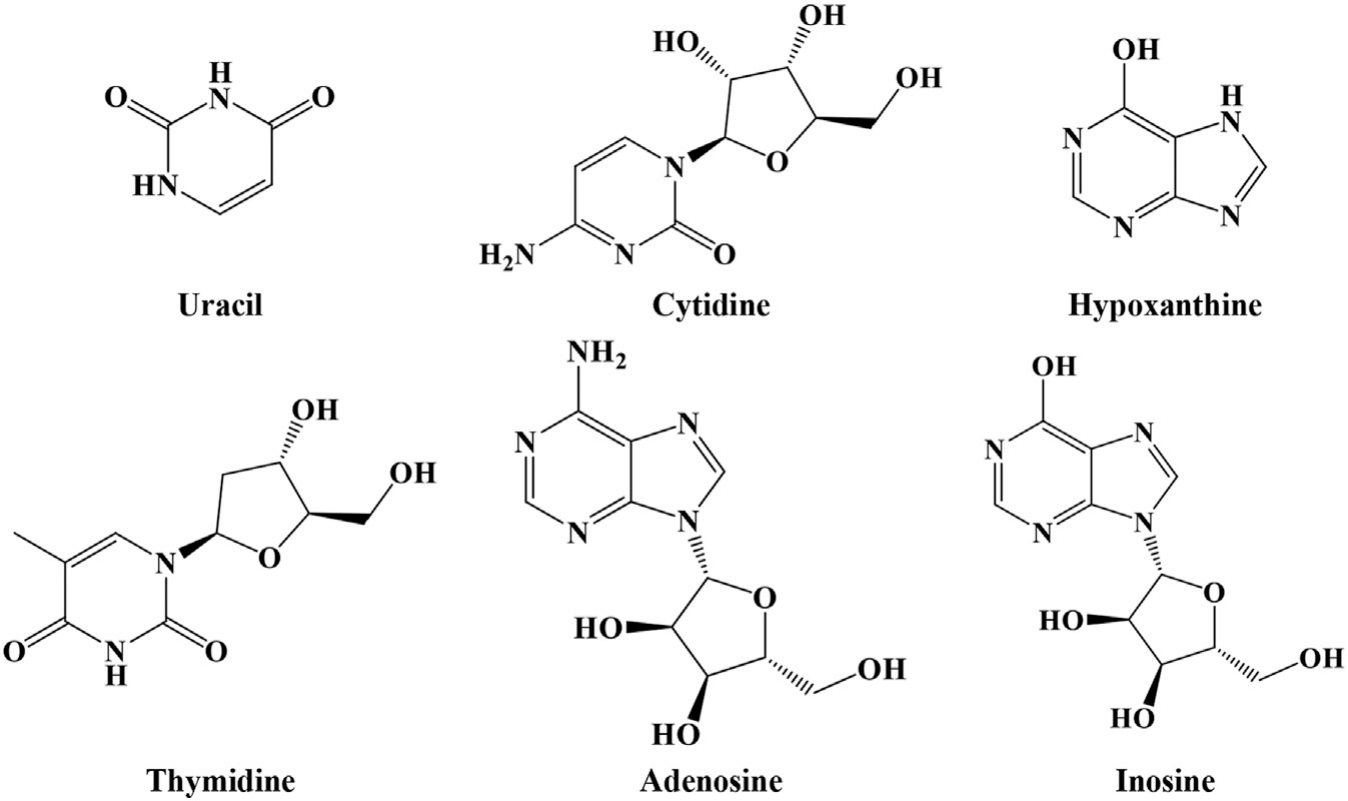
Structures of nucleosides from IN.

As shown in Figure 5, IN also includes nucleosides such as uracil, cytidine, hypoxanthine, thymidine, adenosine, and inosine (Lin et al., 2018).

#### Other Compounds

**FIGURE 6:**
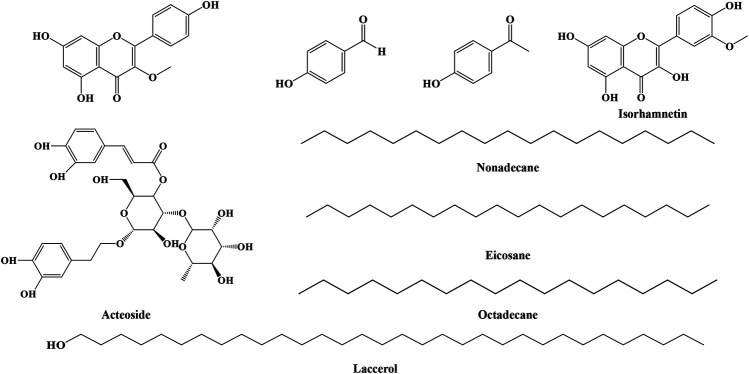
Structures of other compounds from IN.

In addition to the compounds described above, IN contains nine compounds, as shown in Figure 6, such as nonadecane, eicosane, octadecane, laccerol, p-hydroxybenzaldehyde, p-hydroxyacetophenone (Lee et al., 2019), 5,7,4′-trihydroxy-methoxyflavone, isorhamnetin, and acteoside (Wu et al., 2016), which are hard to categorize in our review. IN also contains eighteen kinds of amino acids. Furthermore, IN is rich in inorganic components such as calcium carbonate and silica (Wang, 2004).

### Pharmacokinetics

Only three chemical constituents (indirubin, tryptanthrin, and isatin) have been studied in terms of absorption, distribution, metabolism, and excretion in rats, mice, or beagle dogs. The pharmacokinetic parameters of indirubin, tryptanthrin, and isatin were investigated in three studies (Ren et al., 2016; Jähne et al., 2016; Deng et al., 2008) and are summarized in Table 1. The half-life of indirubin and the area under the curve of indirubin reported by Deng et al. were 35 min and 295 μg h/L, while those reported by Evelyn A were 1 h and 737 μg h/L. The difference may be explained by the variability of cytochrome P450 isozyme expression in the two strains, different administered doses, body weights, extraction of plasma samples, and chromatographic conditions. The absorption process of indirubin in the body through intravenous administration was in accordance with linear kinetics. The lower bioavailability of indirubin when intraperitoneally injected may be related to the first-pass hepatic effect. On the one hand, indirubin is metabolized by CYP1A1, CYP1A2, or CYP1B1. The possible metabolite of indirubin was indigo carmine. On the other hand, indirubin could induce CYP1A1, CYP1A2, or CYP1B1 CYP enzymes and thus accelerate their own metabolism (Spink et al., 2003; Adachi et al., 2004; Kuriyama et al., 2004).

**TABLE 1:** Pharmacokinetics of indirubin, isatin, and tryptanthrin in IN.

Included study	Methods	Animals	Interventions	T_1/2_(h)	T_max_(h)	CL/F (L/h/kg)	V/F (L/kg)	Cmax (μg/L)	AUC_0-t_ (μg h/L)	AUC0-∞ (μg h/L)
Deng et al. (2008)	HPLC-UV	6 male Wistar rats	Indirubin, 5.6 mg/kg, i.v	1.030 ± 0.2	0.017	N/A	N/A	201 ± 23.7	295 ± 45.2	308 ± 50.0
Deng et al. (2008)	HPLC-UV	6 male Wistar rats	Indirubin, 2.8 mg/kg, i.v	1.020 ± 0.2	0.017	N/A	N/A	155 ± 17.7	124 ± 43.3	130 ± 48.3
Deng et al. (2008)	HPLC-UV	6 male Wistar rats	Indirubin, 5.6 mg/kg, i.p	1.080 ± 0.4	0.010	N/A	N/A	20.8 ± 7.6	22.6 ± 5.2	25.9 ± 4.9
Jähne et al. (2016)	UPLC-MS/MS	Male SD rats	Indirubin 2 mg/kg, i.v	0.583 ± 0.07	0.083	2.71 ± 0.52	2.25 ± 0.296	811 ± 140	737 ± 190	763 ± 177
Ren et al. (2016)	Lc- (MS)/MS	Male beagles	Isatin, 15 mg/kg, i.v	0.89 ± 0.24	N/A	6.57 ± 1.98	7.98 ± 0.34	N/A	2,391 ± 669	2,418 ± 675
Ren et al. (2016)	Lc- (MS)/MS	Female beagles	Isatin, 15 mg/kg, i.v	1.13 ± 0.47	N/A	6.72 ± 1.41	11.49 ± 7.19	N/A	2,260 ± 446	2,293 ± 438
Ren et al. (2016)	Lc- (MS)/MS	Male beagles	Isatin, 15 mg/kg, i.g	0.71 ± 0.10	0.67 ± 0.29	N/A	N/A	634 ± 253	1,012 ± 466	1,031 ± 459
Ren et al. (2016)	Lc- (MS)/MS	Female beagles	Isatin, 15 mg/kg, i.g	0.95 ± 0.15	0.67 ± 0.29	N/A	N/A	619 ± 152	922 ± 161	937 ± 163
Ren et al. (2016)	Lc- (MS)/MS	Male beagles	Isatin, 30 mg/kg, i.g	1.19 ± 0.19	0.83 ± 0.29	N/A	N/A	1,902 ± 357	3,578 ± 553	3,624 ± 541
Ren et al. (2016)	Lc- (MS)/MS	Female beagles	Isatin, 30 mg/kg, i.g	1.20 ± 0.36	0.50 ± 0.00	N/A	N/A	2,213 ± 347	3,184 ± 128	3,212 ± 128
Ren et al. (2016)	Lc- (MS)/MS	Male beagles	Isatin, 60 mg/kg, i.g	1.04 ± 0.09	0.83 ± 0.29	N/A	N/A	4,812 ± 412	8,071 ± 1,464	8,164 ± 1,509
Ren et al. (2016)	Lc- (MS)/MS	Female beagles	Isatin, 60 mg/kg, i.g	1.70 ± 0.89	1.00 ± 0.00	N/A	N/A	3,891 ± 284	6,748 ± 927	6,793 ± 923
Jähne et al. (2016)	UPLC-MS/MS	Male S-D rats	Tryptanthrin 2 mg/kg i.v	0.677 ± 0.111	0.071 ± 0.025	1.00 ± 0.36	1.02 ± 0.53	3,526 ± 755	2082 ± 683	2,228 ± 877

Tryptanthrin showed high permeability across human colonic adenocarcinoma cells and the blood–brain barrier. Its transport was not affected by P-gp (Jähne et al., 2016). Zhang et al. (2014) found that tryptanthrin had the highest concentration in the liver, followed by the kidney, and could not be detected in the brain. The differences in the properties demonstrated by indirubin when crossing the blood–brain barrier *in vitro* and *in vivo* should be further explored. Tryptanthrin may be metabolized to a monohydroxylated form by the cytochrome P450 enzyme (Lee et al., 2007). Isatin was quickly absorbed into the body in beagles and then eliminated rapidly. The elimination of isatin in beagles showed no significant gender difference. There was no linear relationship between dose and C_max_ or AUC_0-t_ values (Ren et al., 2016).

### Pharmacology

#### Antitumor Effect

The active components of indirubin, tryptanthrin, and isorhamnetin in IN showed antitumor effects. Tryptanthrin could significantly reduce the size and multiplicity of skin tumors. In addition, tryptanthrin impeded the diffusion of hair follicle cells by interfering with the activity of β-catenin and inhibited A431 cells by abolishing the activation of β-catenin (Shankar et al., 2019). Moreover, the oxygen atom at position 6 of the tryptanthrin ring was coupled to heme iron of tryptophan 2,3-dioxygenase, which was a therapeutic target in cancer diseases, via coordination interaction and thus exhibited higher tryptophan 2,3-dioxygenase inhibitory activity (Zhang et al., 2018). Indirubin was used as a new agent to treat leukemia in China in the 1970s. The antileukemic effect of indirubin was related to the inhibition of microtubule polymerization. Indirubin interacted with amino acid residues at the interface of the α–β tubulin heterodimer *via* electrostatic and hydrophobic interactions and thus interfered with microtubule polymerization. Indirubin showed excellent inhibitory potential against CDKs, which were promising targets for therapeutic intervention in many tumor cells. The formation of the three hydrogen bonds between indirubin and amino residues of Glu81, Phe82, and Leu83 of CDK helped to bind indirubin to the ATP binding site of CDK (Eisenbrand et al., 2004; Mohan et al., 2018). Tryptanthrin and isorhamnetin not only promoted apoptosis but also inhibited the activity of K562 cells. The mechanism by which isorhamnetin treats leukemia is mediated by targeting proto-oncogene tyrosine protein kinase through the cell cycle of K562 cells (Wu et al., 2016).

#### Antiangiogenic Effect

Both IN and tryptanthrin restrained angiogenesis *in vivo* and vascular endothelial cells *in vitro*. Tryptanthrin exerted an antiangiogenic effect through several aspects. First, tryptanthrin regulated the cell cycle in association with cyclins and CDKs. Furthermore, tryptanthrin prevented tube formation and cell migration. Moreover, tryptanthrin suppressed FAK and the PKB pathway (Chang et al., 2015). Additionally, the mRNA stability and promoter activity of apelin were downregulated by tryptanthrin (Chang et al., 2019). VEGFR2 regulated angiogenesis; tryptanthrin could bind to amino acid residues including Cys917 and Glu883 through hydrogen bond or Van der Waals force in the active site of VEGFR2 and exhibit a restrained effect on VEGFR2 (Liao et al., 2013).

#### Anti-Inflammatory Effect

Different active components in IN have shown anti-inflammatory effects through different mechanisms. IN was dissolved in dimethyl sulfoxide, and IN impeded O^2•−^ production and elastase release by inhibiting the activation of MAPK and regulating calcium mobilization in human neutrophils. However, indigo, indirubin, and tryptanthrin had no similar effect (Lin et al., 2009). IN exerted anti-inflammatory effects by reducing the adhesion of lymphocytes to HUVECs induced by TNF-a. Furthermore, IN restrained the expression of VCAM-1 by inhibiting AP-1, which is an agonist of VCAM-1 expression (Chang et al., 2010). Both IN and indigo promoted the expression of IL-10 and IL-22 (Kawai et al., 2017). IN also ameliorated inflammation by downregulating pro-inflammatory factors such as IL-1α, IL-1β, IL-6, IL-8, TNF-α, and IL-18 (Wang et al., 2017). Indirubin could reduce inflammatory reactions by suppressing γδ T cells and inhibiting the activation of Jak3/Stat3 pathways. Indirubin and indigodole A suppressed the gene expression of IL-17 in a dose-dependent manner and demonstrated no cytotoxicity toward Th17 and Jurkat cells. Tryptanthrin and indigodole C inhibited the production of IL-17 in Th17 cells (Lee et al., 2019).

#### Antimicrobial Effect

Twenty-five grams of IN was extracted with 300 ml of ethyl acetate at 40°C for 1 h. The ethyl acetate extracts of IN exerted remarkable inhibitory effects on *Staphylococcus aureus* and *S. epidermidis* but slight effects on *Aspergillus fumigates* and *Candida albicans*. Isatin and tryptanthrin were the active ingredients against *Staphylococcus aureus* and *Staphylococcus epidermidis*. Tryptanthrin played a role in bacteriostatic action due to its binding to DNA of pathogenic organisms *via* intercalation and altered DNA supercoiling (Bandekar et al., 2010). The components inhibiting methicillin-resistant *S. aureus* should be further identified (Chiang et al., 2013).

#### Anti-Psoriatic Effect

Claudin-1 was upregulated and function of TJ was restored in keratinocytes after IN treatment. The major active ingredients, such as indigo, indirubin, and tryptanthrin, exhibited cooperative effects to repair the functions of TJ (Lin et al., 2013). In addition, indirubin exhibited anti-psoriatic effects by regulating the propagation and differentiation of keratinocytes (Lin et al., 2007; Lin et al., 2009). Indirubin also inhibited the proliferation of keratinocytes by suppressing CDC25B expression. In addition, tryptanthrin could downregulate the IL-17 pathway, which was upregulated in the pathological process of psoriasis (Cheng et al., 2017).

#### Influence on the Microbiota

At the phylum level, IN administration increased the proportion of *Firmicutes* and decreased the proportion of *Bacteroidetes* compared with the control group. At the family level, IN treatment showed a two-fold increase in the proportion of *Bifidobacteriaceae* and *Ruminococcaceae* vs. DSS-treated rats, which were the major butyrate producers. At the genus level, IN increased the proportion of beneficial bacteria such as *Ruminococcus_1, Ruminococcaceae_UCG-005, norank_f__Erysipelotrichaceae, Butyricicoccus*, and *Bifidobacterium* while decreasing the harmful bacterium *Escherichia–Shigella* (Sun Z. et al., 2020)*.* Moreover, IN increased the percentage of *Peptococcus* and decreased the percentage of *Turicibacter* (Liang et al., 2019). The pharmacological activities of IN and its compounds are listed in Table 2.

**TABLE 2:** Pharmacological activities of IN and its compounds.

Drug	Doses	Study	Experimental model	Effects	References
**Antitumor effect**					
Isorhamnetin	12.5, 25, and 50 μM	*In vitro*	K562 cells	Inducing G2/M cell cycle arrest by targeting Src	Wu et al. (2016)
Tryptanthrin	0.5 and 1 mg	*In vivo*	DMBA/PMA-induced skin cancer in female Swiss albino mice	Significantly reducing the tumor size and multiplicity, suppressing the activation of β-catenin	Shankar et al. (2019)
Tryptanthrin	3, 6, 9, and 12 μmol/L	*In vitro*	A431	Inhibiting the proliferation of the cells, suppressing the activation of β-catenin	Shankar et al. (2019)
**Antiangiogenic effect**					
IN	10, 50, 250, 500, and 1,000 μg/ml	*In vitro*	VEGF induced HVECs angiogenesis	Inhibiting the cell proliferation of HVECs	Chang et al. (2015)
Tryptanthrin	10, 25, and 50 μM	*In vitro*	VEGF induced HVECs angiogenesis	Suppressing the cell cycle progression, cell migration and tube formation through PKB and FAK pathway	Chang et al. (2015)
Tryptanthrin	10, 25, and 50 μM	*In vitro*	VEGF human HVECs angiogenesis	Inhibiting the expression of apelin	Chang et al. (2019)
**Anti-inflammatory effect**					
IN	250 and 500 μg/ml	*In vitro*	HUVECs	Reducing lymphocyte adhesion to HUVECs and suppressing the expression levels of VCAM-1	Chang et al. (2010)
IN	4.2, 8.4, and 16.8 g/kg	*In vivo*	3.5% DSS induced UC in Sprague–Dawley rats	Reducing the expression of inflammatory cytokines such as IL-1α IL-1β and IL-18	Wang et al. (2017)
IN	0.3, 1, 3, and 10 μg/ml	*In vitro*	FMLP induced human neutrophils activation	Impeding O2•− production and elastase release by inhibiting the activation of MAPK and regulating calcium mobilization	Lin et al. (2009)
**Antimicrobial effect**					
Ethyl acetate extract of IN	1, 2, 3, and 4 mg/disc	*In vitro*	Bacterial and fungal strains	Inhibiting Gram-positive bacteria and non-dermatophytic onychomycosis pathogens	Chiang et al. (2013)
**Anti-psoriatic effect**					
IN	10, 50, 250, and 500 mg/ml	*In vitro*	Human keratinocytes	Suppressing the expression of PCNA, increasing the expression of involucrin, increasing the activity of PKC, upregulating the claudin-1 expression and restoring the tight junction function	Lin et al. (2009), Lin et al. (2007)
Indirubin	5, 10, 50, and 100 μM	*In vitro*	Human keratinocytes	Suppressing the expression of PCNA, increasing the expression of involucrin, increasing the activity of PKC, upregulating the claudin-1 expression and restoring the tight junction function	Lin et al. (2009), Lin et al. (2007)
**Influence on the microbiota**					
IN	600 mg/kg	*In vivo*	4.5% DSS induced UC in male Sprague-Dawley rats	Increasing the levels of feces butyrate, the proportion of Ruminococcus_1 and Butyricicoccus	Sun Z. et al. (2020)
IN	100, 200, and 400 mg/kg	*in vivo*	3% DSS induced UC in male Kunming mice	Reducing the relative quantity of Turicibacter and increading the relative quantity of Peptococcus	Liang et al. (2019)

### Clinical Applications

#### Treatment of Ulcerative Colitis

Suzuki et al. first published a report about the efficacy of oral IN in UC patients. Nine intractable UC patients who did not achieve relief after drug interventions, such as amino salicylates, sulfasalazine, 5-aminosalicylic acid, corticosteroids, and azathioprine, using IN were investigated in a retrospective study. The symptoms were improved dramatically in most patients after 4 months of treatment (Suzuki et al., 2013). In 2016, Sugimoto et al. conducted a single-center open-label prospective study using IN for patients with mild to moderate UC activity. Twenty patients received IN orally in a capsule form at a daily dose of 2 g for 2 months. The percentages of clinical response, remission, and mucosal healing were 72, 33, and 61%, respectively. The clinical scores were also significantly improved after IN treatment compared to baseline (Sugimoto et al., 2016). In addition, Naganuma et al. carried out a multicenter, double-blind trial evaluating the safety and efficacy of IN in active UC patients. Eighty-six patients were randomized into four groups and given IN at a daily dose ranging from 0.5 to 2 g for 8 weeks. The results showed that there was a linear trend between clinical responses and daily dose. The highest clinical response was 81% among patients taking 2 g of daily IN. Clinical remission was 55.0% for patients who received 1.0 g of IN and 38.1% for patients who received 2.0 g of IN. These responses were significantly higher than that of the placebo group (4.5%). The mucosal healing rates of patients taking IN were significantly higher than those of the placebo group. Among them, patients taking 1 g of IN had the best results (Naganuma et al., 2018). Then, a subanalysis of Naganuma et al.’s research was conducted to survey whether IN was useful even in patients who were dependent on steroids and refractory to anti-TNF. The results showed that IN was effective even in refractory patients (Naganuma et al., 2020). In previous studies, we determined that the clinical effect of IN in treating UC is remarkable. However, the efficacy of IN in terms of maintenance treatment in UC is unclear. Matsuno et al. (2020) confirmed that the therapeutic efficacy of IN in UC was favorable, whereas in Crohn’s disease, it was modest.

#### Treatment of Psoriasis

The efficacy of IN in the treatment of psoriasis through oral administration has been demonstrated in several clinical trials. However, poor solubility and absorption coupled with frequent adverse gastrointestinal reactions and hepatotoxic effects of IN restrict its clinical use. To preserve the efficacy of IN and avoid systemic adverse effects, Lin et al. (2007) developed IN ointment which consisted of 20% IN and 45% olive oil for topical application in patients with recalcitrant plaque psoriasis. Fourteen patients were topically administered either vehicle or IN ointment for 8 weeks. The results suggested that topically applied IN ointment significantly reduced clinical scores and improved skin histology. In 2008, Lin et al. carried out a randomized, observer-blind, vehicle-controlled, interpatient comparison study that included more patients than their previous study. Forty-two outpatients topically applied either vehicle or IN ointment to symmetrical psoriasis lesions. Psoriasis was cleared or nearly cleared after IN ointment treatment in 74% of patients after 12 weeks. Although IN oil extract had a remarkable therapeutic effect in treating nail psoriasis without adverse effects in many patients, it had a negative effect on compliance because of difficulty in cleaning and aesthetically unappealing dark blue stains on nails. Lin et al. (2011) further developed a refined formulation named Lindioil to improve patient compliance and evaluated the therapeutic effect of this refined formulation in treating psoriatic nails (Lin et al., 2011). Twenty-eight of thirty-two patients with nail psoriasis received Lindioil for 24 weeks. The mean NAPSI and modified target NAPSI were markedly reduced compared to baseline at the end of the study, although this trial lacked a comparison. Consequently, a randomized, observer-blind, vehicle-controlled, and intrasubject trial was conducted by Lin et al. The therapeutic effect of Lindioil was significantly better than that of the control group. No adverse events occurred during the treatment (Lin et al., 2014). Indirubin is the main active component of Lindioil. Lin et al. further compared the efficacy and safety of Lindioil, which contains different concentrations of indirubin, in a randomized, double-blind trial. Lindioil containing 10 μg/g, 50 μg/g, 100 μg/g, and 200 μg/g indirubin was applied to adult patients for 8 weeks. An additional 12 weeks were included for the subjects as a safety/extension period. The results showed that 200 μg/g indirubin was the most effective concentration and safe for topically treating psoriasis (Lin et al., 2018). A summary of clinical trials supporting the application of IN is given in Table 3.

**TABLE 3:** Summary of clinical trials supporting the application of IN.

Disease	Study design/sample size	Treatment	Outcomes	Reference
UC	Placebo-controlled RCT/86	0.5 g IN, once daily, orally for 8 weeks	The rate of clinical response: 69.6% (0.5 g IN), 13.6% (placebo)	Naganuma et al. (2018)
		1 g IN, once daily, orally for 8 weeks	The rate of clinical response: 75% (1.0 g IN), 13.6% (placebo)	
		2 g IN, once daily, orally for 8 weeks	The rate of clinical response: 81% (2.0 g IN), 13.6% (placebo)	
		0.5 IN, once daily, orally for 8 weeks	The rate of clinical remission: 26.1% (0.5 g IN), 4.5% (placebo)	
		1 g IN, once daily, orally for 8 weeks	The rate of clinical remission: 55% (1.0 g IN) vs. 4.5% (placebo)	
		2 g IN, once daily, orally for 8 weeks	The rate of clinical remission: 38.1% (2.0 g IN) vs. 4.5% (placebo)	
		0.5 IN, once daily, orally for 8 weeks	The rate of mucosal healing: 56.5% (0.5 g IN), 13.6% (placebo)	
		1 g IN, once daily, orally for 8 weeks	The rate of mucosal healing: 60% (1.0 g IN), 13.6% (placebo)	
		2 g IN, once daily, orally for 8 weeks	The rate of mucosal healing: 47.6% (2.0 g IN), 13.6% (placebo)	
UC	Open-label, prospective pilot/20	2 g IN, once daily, orally for 8 weeks	The rate of clinical response: 72%	Sugimoto et al. (2016)
		2 g IN, once daily, orally for 8 weeks	The rate of clinical remission: 33%	
		2 g IN, once daily, orally for 8 weeks	The rate of mucosal healing: 61%	
Psoriasis	Randomized, observer-blind, vehicle controlled trial/42	IN ointment, once daily, topically for 12 weeks	The sum of scaling, erythema, and induration scores: 6.3 (IN ointment), 12.8(vehicle ointment). Plaque area percentage: 38.5% (IN ointment), 90% (vehicle ointment)	Lin et al. (2008)
Psoriasis	Noncontrolled pilot Study/28	IN ointment, twice daily, topically for 24 weeks	The Nail Psoriasis Severity Index: 14.9 ± 11.1 (at week 24), 36.1 ± 14.7 (at baseline)	Lin et al. (2011)
Psoriasis	Randomized, observer-blind, vehicle-controlled trial/31	IN ointment, twice daily, topically for 24 weeks	The reduction of Nail Psoriasis Severity Index: 49.8% (Lindioil group), 22.9% (control group)	Lin et al. (2014)
Psoriasis	Randomized, double-blind, dosage-controlled trial/98	0.5 g IN ointment (200 μg/g of indirubin)/100 cm^2^ of psoriatic lesion area, twice daily, topically for 8 weeks	The reduction of Psoriasis Area and Severity Index: 69.2%, the proportion of subjects achieving 75 and 90% reductions in PASI scores 56·5% and 30·4%	Lin et al. (2018)
		0.5 g IN ointment (100 μg/g of indirubin)/100 cm^2^ of psoriatic lesion area, twice daily, topically for 8 weeks	The reduction of Psoriasis Area and Severity Index: 63.1%, the proportion of subjects achieving 75 and 90% reductions in PASI scores 44 and 8%	
		0.5 g IN ointment (50 μg/g of indirubin)/100 cm^2^ of psoriatic lesion area, twice daily, topically for 8 weeks	The reduction of Psoriasis Area and Severity Index: 50.3%, the proportion of subjects achieving 75 and 90% reductions in PASI scores 24 and 4%	
		0.5 g IN ointment (10 μg/g of indirubin)/100 cm^2^ of psoriatic lesion area, twice daily, topically for 8 weeks	The reduction of Psoriasis Area and Severity Index: 53.4%, the proportion of subjects achieving 75 and 90% reductions in PASI scores 24 and 4%	

#### Adverse Events

Although the clinical efficacy of IN was confirmed, adverse events (AEs) associated with IN should not be ignored. Adverse reactions of IN for external use were minor and mainly occurred through oral administration. These AEs could be categorized into mild AEs, such as liver dysfunction, renal dysfunction, headache, gastrointestinal symptoms, and nausea, and severe AEs, such as acute colitis, colonic intussusception, and pulmonary arterial hypertension (PAH).

#### Liver Dysfunction

Several studies have reported liver dysfunction of IN in treating UC. Liver dysfunction occurred even at a low daily dose of 0.5 g (Naganuma et al., 2018). Transaminase levels returned to normal levels in most patients without discontinued use of IN. There were no cases involving the development of fulminant hepatitis or requiring hospitalization in patients with liver dysfunction. Liver dysfunction associated with IN tends to be reversible and mild (Sugimoto et al., 2016; Naganuma et al., 2019; Matsuno et al., 2020). CYP1A1 was induced by IN *via* AhR, which was hyperactive in the liver and may be associated with liver dysfunction. Although the reason that IN caused liver dysfunction was not fully clear, liver function should be monitored in the course of treatment.

#### Renal Dysfunction

Renal dysfunction was reported in a retrospective observational study. Serum creatinine levels in patients taking 1 g of IN increased slightly at 28 weeks but exceeded the normal range at 104 weeks. Serum creatinine levels did not return to normal levels as the daily dose decreased from 1 to 0.3 g (Matsuno et al., 2020).

### Pulmonary Arterial Hypertension

A 45-year-old woman who used 2 g of self-purchased IN daily for 6 months showed symptoms of increasing dyspnea, chest oppression, and leg edema and developed PAH (Nishio et al., 2016). In Japan, 11 of approximately 5,000 patients who used IN to treat UC or Crohn’s disease developed PAH, and they improved by discontinuing IN (Nishio et al., 2018). Although PAH triggered by IN may be reversible, we should be careful with long-term or high-dose IN. In contrast, PAH was not discovered in patients who received IN or placebo for the treatment of UC for eight weeks in a multicenter randomized controlled trial. PAH was not observed during the 8- to 16-month follow-up period in any patients after study termination (Naganuma et al., 2018). Surveillance of PAH induced by IN should be continued in the future. The causal relationship between PAH and IN should be further investigated.

#### Acute Colitis

Acute colitis was a serious adverse event involving abdominal pain with bloody diarrhea, wall thickening, and edema in patients who required cessation of IN or even hospitalization. Two patients reported by Kondo et al. developed colitis during the treatment of UC through oral IN with dosages ranging from 1 to 2 g within three months. They were taking no other medicines. Their symptoms improved after the cessation of IN (Kondo et al., 2018). In addition, one patient developed acute colitis after the patient increased the daily dosage of IN from 1 to 3.5 g on their own (Yanai et al., 2018). The patients experienced relapse after the cessation of IN. However, acute colitis did not recur in subjects reported by Matsuno et al. (2020) after the resumption of IN.

#### Intussusception

In a Japanese nationwide survey, ten cases of intussusception occurred within 2 months. The range of daily dosages of IN in six patients was 0.5–2.0 g. The cecum and/or ascending colon was the site of intussusception in all patients, and forty percent of these patients were required to undergo surgical resection (Naganuma et al., 2019).

## Quality Control

Sources of IN include Strobilanthes cusia (Nees) Kuntze, Persicaria tinctoria (Aiton) Spach, and Isatis tinctoria L*.* at different harvest times and geographical conditions, and the processing of IN might lead to differences in the inherent quality of IN. A total of twelve organic compounds, specifically indirubin, indigo, tryptanthrin, isatin, indoxyl β-D-glucoside, indole, indole-3-aldehyde, anthranilic acid, β-sitosterol, betulin, daucosterol, and isorhamnetin, and two inorganic compounds, calcium carbonate and silica, have been quantified by different research groups. The results of the quantitative analysis of IN are listed in Table 4 (Plitzko et al., 2009; Wu et al., 2016; Naganuma et al., 2018; Pan et al., 2018; Sun Z. et al., 2020; Liang et al., 2019).

**TABLE 4:** Quantitative analysis for the quality control of IN.

Analyte	Method	Results	References
Indigo	1H-NMR	1.4–2.7%	Plitzko et al. (2009)
Indigo	HPLC	1.1–1.4%	Plitzko et al. (2009)
Indirubin	HPLC	0.16–0.37%	Plitzko et al. (2009)
Indigo	HPLC	1.18–3.47%	Pan et al. (2018)
Indirubin	HPLC	0.09–0.26%	Pan et al. (2018)
Indigo	FTIR spectroscopy	1.46–2.82%	Pan et al. (2018)
Indirubin	FTIR spectroscopy	0.12–0.23%	Pan et al. (2018)
CaCO_3_	ICP-OES	59.125–96.175%	Pan et al. (2018)
CaCO_3_	FTIR spectroscopy	60.925–87.125%	Pan et al. (2018)
Indigo	HPLC	30.07%	Sun Z. et al. (2020)
Indirubin	HPLC	0.145%	Sun Z. et al. (2020)
Indigo	UPLC	13.87%	Liang et al. (2019)
Indirubin	UPLC	0.15%	Liang et al. (2019)
Indole	UPLC-APCI-TOFMS	0.0002574%	Naganuma et al. (2018)
Indole-3-aldehyde	UPLC-APCI-TOFMS	0.0000203%	Naganuma et al. (2018)
Tryptanthrin	UPLC-APCI-TOFMS	0.000124%	Naganuma et al. (2018)
Indigo	UPLC-APCI-TOFMS	0.2507%	Naganuma et al. (2018)
Indirubin	UPLC-APCI-TOFMS	0.08347%	Naganuma et al. (2018)
Betulin	UPLC-APCI-TOFMS	0.0007956%	Naganuma et al. (2018)
β-Sitosterol	UPLC-APCI-TOFMS	0.003974%	Naganuma et al. (2018)
Daucosterol	UPLC-APCI-TOFMS	0.0009216%	Naganuma et al. (2018)
Indican	UPLC-ESI-TOFMS	0.00001328%	Naganuma et al. (2018)
Anthranilic acid	UPLC-ESI -TOFMS	0.001356%	Naganuma et al. (2018)
Indigo	Q-TOFMS	13.81%	Wu et al. (2016)
Indirubin	Q-TOFMS	3.7%	Wu et al. (2016)
Tryptanthrin	Q-TOFMS	0.72%	Wu et al. (2016)
Isorhamnetin	Q-TOFMS	0.04%	Wu et al. (2016)
CaCO_3_	Titration	68.51%–71.50%	Wang (2004)
Silica	Titration	7.78–11.09%	Wang (2004)

## Discussion

Although IN has achieved good curative effects in clinical applications, adverse reactions are obstacles to its clinical application. A clinical trial investigating the efficacy of IN in UC patients was stopped because of a report of a patient who used IN for six months who developed PAH. The adverse reactions of IN were classified into moderate and severe adverse reactions. Some serious adverse reactions, such as PAH and intussusception, even required hospitalization or surgery. The adverse reactions of IN for topical use were mild and mainly occurred in the course of oral administration within the therapeutic dose range of 0.5–3 g. Benefit–risk assessment should be performed during treatment. At the same time, the mechanism of adverse reactions should be further studied.

The presence of indigo and indirubin in human urine suggests that some chemical components in IN were endogenous active ingredients. This was because tryptophan obtained from food or broken down by proteins in the body was degraded to indigo or indirubin through a series of chemical reactions by intestinal bacteria and enzymes. The interference of endogenous active ingredients should be excluded in the pharmacokinetic study of IN. The pharmacokinetics of IN involve only a few active components. Further studies including more active ingredients should be carried out to obtain the overall pharmacokinetic characteristics of IN and provide a basis for the study of pharmacological action and adverse reactions as well as drug delivery systems.

The content of active ingredients such as indigo and indirubin in IN varies greatly in different studies, ranging from 0.2507 to 30.07% and from 0.08347 to 3.7%, respectively. There are several reasons for this variation. First, the content of active ingredients of the herb medicine is different because of the origin, harvest time, and processing. Second, different extraction solvents, such as methanol, CHCl_3_/MeOH (1:1), DMSO-d6, N, N-dimethylformamide, and CHCl_3_, with 2% chloral hydrate used in the sample preparation have an effect on the inconsistency of the results. Third, different chromatographic methods may also lead to these differences. Thus, developing a standardized quality control method for IN is urgent and indispensable. In addition, the control of a few components, such as indigo or indirubin, makes it difficult to determine the quality of IN.

IN is a multicomponent herbal medicine containing both organic and inorganic compounds that shows inhibitory effects on inflammation, tumors, bacteria, and psoriasis. More importantly, IN is effective in the treatment of UC and psoriasis based on randomized, double-blind controlled clinical trials. However, the reason why IN has good clinical effects is not entirely clear. In the future, we should further carry out pharmacological research on chemical compounds that have not been found or have synergistic effects in IN. In addition, we can conduct multicomponent pharmacokinetic studies of IN, which could guide clinicians to use drugs effectively and safely. At the same time, multi-omics techniques can be applied to research the pharmacological mechanism of IN.

## Abbreviations

IN: indigo naturalis, UC: ulcerative colitis, CYP: cytochrome p450, PAH: pulmonary arterial hypertension, AhR: aryl hydrocarbon receptor, VEGF: vascular endothelial growth factor, A431: human epidermoid carcinoma cell line, HVECs: human vascular endothelial cells, PKB: protein kinase B, FAK: focal adhesion kinase, HUVECs: human umbilical vein endothelial cells, VCAM-1: vascular cell adhesion molecule-1, EGF: epidermal growth factor, DAI: disease activity index, MAPK: mitogen-activated protein kinase, Src: proto-oncogene tyrosine-protein kinase, PCNA: proliferating cell nuclear antigen, PKC: protein kinase C, FMLP: formyl-l-methionyl-l-leucyl-lphenylalanine, TNF-a: tumor necrosis factor-a, AP-1: activator protein-1, K562: erythroleukemia cell line, CDK: cyclin-dependent kinase, IL-17 cells: IL-17-producing T helper cells, Jak3: Janus kinase 3, Stat3: signal transduction and activation of transcription 3, TJ: tight junction.
